# Medical Opinion and Movement

**Published:** 1908-09-19

**Authors:** 


### Medical Opinion and Movement.

Dr. KONOSSEVITCH (*Roussky Vratch*) draws attention to his success in treating certain extremely chronic cases of eczema by means of the actual cautery applied over the region of the spine. His cases numbered twelve, and in each the eczematous condition was generalised. Five of the twelve cases were for various reasons lost sight of, but the remaining seven showed a cure which was continued for a long time. Two or three applications of the cautery were given in each case, the needle being applied in six or eight different spots.

M. KROMER recently communicated to the Berlin Society of Medicine the result of operations of pubiotomy carried out in Bumm's clinic since 1905. Out of 53 cases operated upon, death resulted in one case. As regards the infants, 92 per cent. were born alive, and 86 per cent. were alive when their mothers left the clinic. In over half the cases delivery was followed by fever, but this complication, as well as those caused by lesions of the soft parts and urinary passages, in no way prevented recovery. In five cases intervention was necessary on account of urinary fistulæ. Subsequently nine of the women again became pregnant, and in eight of these delivery was accomplished by natural means. In the ninth case a second pubiotomy on the opposite side was performed. Only in five cases out of 34 examined did bony union occur after operation. It must, moreover, be noted that about half of the patients operated upon suffered a certain amount of disability after pubiotomy. Two complained of being unable to lift heavy objects, but most complained of some genito-urinary trouble or other; in 15 cases retention of urine was complained of, and in 13 others uterine retroflexion was found.

SOME recent experiments of Tchistovitch and Jourevitch (*Annales de l'Institut Pasteur*) would seem to throw light upon the mechanism of cure in cases of pneumococcal infection. As long ago as 1890 the first-mentioned observer showed that in cases of infection by diplococci of low virulence an intense phagocytosis and leucocytosis are induced, whereas if the infection is due to virulent organisms leucopenia and an absence of leucocytosis are the result. The same observer later showed that even in the case of strongly immunised animals the diplococci are able to live a long time in the organism if protected from the action of the phagocytes—*e.g.* if introduced into the anterior chamber of the eye of a pigeon, where the leucocytes arrive but slowly. In the case of human beings the same correlation exists between the degree of virulence of the infective organisms and the faculty of reacting towards them by leucocytosis and phagocytosis. At the moment when the crisis occurs the blood of patients suffering from pneumonia possesses no well-marked bactericidal powers. To demonstrate this fact Tchistovitch and Jourevitch have undertaken the following more exact experiments: Into the abdominal cavity of a certain number of dogs small collodion globules containing cultures of diplococci were introduced. At the same time the dogs were infected by injecting into one lung similar cultures of pneumococci. By various ingenious devices the authors arranged that the serum of the animals should be able to reach the cultures in the collodion globules, while the leucocytes were prevented from entering therein. It was found that the dogs stood the pneumococcal infection well, and that after having completely recovered from the disease it was possible to recover from their abdominal cavities diplococci which had retained their virulence. The authors argue from this that diplococci which are quite accessible to the action of the organic fluids of the body are capable of living and retaining their virulence in the body of a dog completely cured of a pneumonia, provided that these diplococci are protected from the action of the phagocytes. Hence these observers conclude that the blood of such animals possesses no bactericidal action of its own as regards the pneumococcus, but depends on the action of the phagocytes in the body to effect a cure.

DR. TRIVAS, of Nevers, publishes in *La Clinique* his observations on the effects of subcutaneous injections of cocaine as a preventive of vomiting after the administration of a general anæsthetic. He has tried the treatment in 112 cases, and the following is the procedure he employs: Half an hour before anæsthesia is induced the patient is given a subcutaneous injection of a solution of morphine and scopolamine; .01 gram of morphine, and .0003 gram scopolamine in the case of an adult man, somewhat smaller doses in the case of a woman. These drugs are given to hasten anæsthesia and shorten the period of excitation. Pure ether is the anæsthetic chosen when the patient has any cardiac lesion, but a mixture of ether and chloroform is given to normal patients. Just before the operation is completed, a subcutaneous injection of cocaine is administered to the patient. In the case of a man, the quantity administered is .025 gram; in that of a woman, .020. The result of these proceedings is that there is a noticeable decrease of post-anæsthetic vomiting. Instead of about 33 per cent. of patients suffering from this trouble, as is, according to the author, usually the case, the percentage is reduced to slightly over 9 per cent., and in most of these cases the vomiting appears much later than usual. Moreover, the cocaine injections make the patients feel more comfortable than is usually the case on regaining consciousness, and the blood-pressure is raised thereby.

AT the annual general meeting of the Deutsche Aerzte Verband, held at Danzig this year, a number of interesting ethical questions were discussed. This Association is a body which was formed some years ago to protect the professional interests of German medical practitioners, and which has consistently shown itself able and willing to deal in a broad-minded and liberal spirit with the many questions which concern the profession to-day. This year several important matters were brought forward, among others the question of the position of practitioners attached to a hospital. This matter had been referred to a special committee, which brought forward a report in which the following recommendations were suggested. That the chief practitioner in a hospital be appointed in terms of a written contract, and that in case he has not the direction of the administrative department of the hospital, he be the chairman of the administrative board; that he be supreme, so far as the clinical department of which he is the head is concerned, and subject to no layman or lay commission. In the case of the assistants, they recommended that these should be appointed only by the chief practitioner, at a salary of at least £60 yearly with an annual increment of £10, with accident insurance privileges from the hospital, and that they should be subordinate only to the chief of their department. In the discussion that followed the reading of the report, some of the speakers pointed out that the position of the assistants in the German asylums left much to be desired. They are overworked, badly lodged, and underpaid, with the result that there was practically no competition for these posts. In the large general hospitals both the chiefs and the assistants were very well off: they hold their posts on fixed contracts, and in accordance with principles such as those recommended by the committee. This is specially the case at the large Hamburg and Berlin institutions. In the smaller general hospitals, on the contrary, there is much that needs amendment. The professional staff is subordinate to a lay director, and cases of injustice and intermeddling frequently occur. The Association, after full discussion, decided to adopt the committee's recommendations and to support these actively.

---

Another interesting point discussed was the relation of practitioners to purely academic institutions, such as the University Krankenkassen or sick clubs. Dr. Sardemann, of Marburg, pointed out that the various students' sick clubs differed from one another in their regulations. The present Krankenkassen are no longer charitable institutions: they are clubs with special privileges and duties. In each case the patients should be made to pay for their treatment, either privately or through their clubs. The academic Krankenkassen should be placed on the same footing as other clubs. Their members, the students, belong to the average middle class, and in some sections (as at Munich) number several thousands. At present the fees they pay are "poor fees," such as are paid for very poor patients. This is a false standpoint, as the majority of student members do not belong to the class of pauper patients, but are able to pay a higher fee. The whole question of academic students' clubs is interesting, and is an important one for the German practitioner. A member of such a club, by paying a small club fee yearly, gains the privilege of free treatment by the local practitioners, who are paid an almost nominal sum by the clubs for such treatment. The point was fully discussed, but the meeting agreed to defer a decision until more information regarding the various clubs was available, and accordingly postponed any final decision until the next general meeting. On the question of the examination of school children the meeting agreed with the recommendations of the committee, which moved that the examining practitioner should be paid at the rate of not less than 0.50 marks per child per year in ordinary cases, and not less than 0.75 marks per child per year in the case of elementary scholars.

---

Although stretching of the sciatic nerve is a recognised form of treatment for obstinate cases of sciatica, it is by no means always successful. Its efficacy was supposed to depend upon some influence which it exercised upon the nerve fibres, but it now appears to be due to the severance of adhesions formed around the nerve trunk as a result of a perineuritis. According to recent reports better results are obtained by exposing the nerve high up close to the great sciatic foramen and dividing the adhesions. It may be remembered that Laségue's sign consists in flexing the thigh on the pelvis with the leg extended, when in cases of sciatica acute pain is caused in the course of the sciatic nerve. Fajersztajn has shown that the same pain, though less intense, is caused when the opposite healthy leg is similarly treated. The explanation offered is that the pain is due to pulling on the adhesions formed around the nerve. When the opposite leg is manipulated the sciatic nerve is stretched, and a pulling force is communicated to the opposite nerve through the spinal roots and meningeal sheaths. Von Baracz, by dissections on the dead body, finds that the sciatic nerve is much more firmly attached at its origin above the sciatic notch than in its course down the leg, and therefore suggested exposure of the nerve high up and separation of the adhesions found in that region. The operation has been carried out by Venglvosky, and more recently by Pers, a Danish surgeon. The latter has reported on 47 cases of sciatica in which he has operated with success. In the first 30 he exposed the nerve by an incision below the gluteal muscles, but in the last 17 cases he carried the incision higher up so as to expose the nerve at the great sciatic notch, according to the method advocated by Baracz. According to Pers adhesions are the most constant change found in cases of sciatica, and in his 47 cases they were absent only in two, so that the affection appears to be essentially a perineuritis. The operation should be carried out rapidly so as to avoid manipulation and exposure of the nerve as far as possible.

---

The operation for excision of cancerous growths of the rectum has undergone considerable evolution in recent years, and in the August issue of the *International Journal of Surgery* Dr. F. G. Du Bose gives an excellent review of the different methods of operating and their results, and describes the surgical procedure which he has adopted and which generally conforms to that now followed by surgeons in this country. He shows that the heavy mortality which has attended this class of operation during the past decade has been largely due to the desire of the surgeon to preserve the original anal outlet for fæcal discharge. Over 50 per cent. of deaths have been due to sepsis, and this serious factor can only be eliminated by freeing the rectum from fæcal contamination by means of a preliminary colotomy. Moreover, it is now recognised that the possibility of a satisfactory anal orifice after excision of the rectum is so remote that it is better to accept the inevitable and provide the patient with a permanent artificial anus by colotomy. The whole procedure, therefore, is carried out in two stages. First, a colotomy operation is performed, the sigmoid is divided between clamps by the thermo-cautery, the lower segment is invaginated and closed by sutures and with the upper segment an artificial anus is provided. When the patient is fully recovered and the rectum rendered as sterile as possible by frequent lavage, the second operation of excision is carried out. The second great danger is that of recurrence, which is noted in 35 per cent. of cases that recover from the operation. Dr. Du Bose recommends that the wound be left open to heal gradually by granulation, and that a daily application of the *x*-rays be administered during the process of healing. In order to give a sphincter-like opening to the artificial anus Dr. Du Bose makes two abdominal incisions parallel to one another and about three inches apart, separates the external oblique muscle from the internal oblique aponeurosis, and draws the upper sigmoid segment through between these and out through the external incision. Thus he claims that the patient has to a large extent voluntary control over his evacuations.

---

Local anæsthesia is now largely used for all manner of minor surgical operations, but it does not appear to have occurred to surgeons to make use of this form of anæsthetic for the reduction and treatment of fractures. M. Quénu, however, has hit upon the happy idea, and at a recent meeting of the Surgical Society in Paris he has reported upon several cases he has treated in this way. He injects two or three centigrammes of cocaine around the seat of fracture, and after an interval of a few minutes he proceeds to reduce the fracture and put the limb up in suitable splints or plaster of Paris. The injections cause a satisfactory relaxation of the muscles and enable the surgeon to manipulate quite painlessly. He has carried out the method in 15 cases of various fractures, and in only one did he fail to obtain proper anæsthesia, which he ascribes to an insufficient amount of cocaine injected. It has become an almost universal custom to administer an anæsthetic for the purpose of reducing a fracture, not only to save the patient unnecessary pain, but also in order to obtain a satisfactory relaxation of the muscles; but this often necessitates delay, either for want of the assistance of a second medical man or on account of the unpreparedness of the patient for an anæsthetic. On the other hand, it is often desirable that reduction of the fracture and fixation of the limb should be effected as soon as possible. Local anæsthesia has therefore material advantages in these cases. Further medical assistance can be dispensed with and the patient requires no previous preparation, such as abstinence from food.

Continuous catheterism of the ureters has been successfully practised by Alberan in the treatment of anal fistula following nephrostomy. More recently Cardenal of Madrid advocates the same line of treatment in operative surgery of the bladder, such as supra-pubic cystotomy for removal of a calculus, or for excision of a growth or an enlarged prostate. When the bladder has been opened above the pubes the author introduces a No. 9 or 10 Charrière into each ureter and then passes the other ends of the catheters along the urethra. In this way the vesical cavity is kept dry and free from urine for the first few days after operation, and among other advantages it is especially claimed that post-operative hæmorrhage can be better controlled by dry packing. The catheters can be retained as long as nine days, and in experiments on animals the author has kept them in position for 16 days without any complication arising. He advises the administration of some urinary antiseptic such as urotropine during the treatment. The permeability of the catheters should be tested from time to time by the injection of a little boric acid solution or a 1-per-cent. solution of nitrate of silver. Blocking of a catheter is generally indicated by feelings of discomfort in the region of the corresponding kidney, and the catheter must then be replaced by another, guided by a mandril, according to the method adopted by Alberan. It is suggested that this method of continuous ureteral catheterism might yield good results also in cases of vesical and vesico-vaginal fistula.

---

De Marchis, of Florence, has lately published a valuable contribution to the study of the so-called blood-cysts of the spleen. His observations were made on two cases, in one of which the cause was probably malaria, while in the other the lesion was due to trauma. In the majority of cases such cysts follow upon an injury to the spleen, and are really hæmatomata encapsuled in its substance. The differential diagnosis is often attended with great difficulties, as they are liable to be confounded with tumours of the left kidney or supra-renal, and the author shows that the old statement—namely, that the position of the colon differentiates such lesions from splenic tumours is not always true. The colon may cover a portion of the splenic tumour, while again a pure kidney growth may be uncovered by the large intestine and lie in direct contact with the abdominal wall. From hydatid cysts such splenic tumours are clinically difficult to differentiate, but in the majority of cases the blood examination is helpful, as in hydatid cysts there is an increase of eosinophile cells in the blood. Clinically such splenic tumours present themselves as enlargements of the spleen, which may reach well below the umbilicus. The enlargement is usually hard, dull on percussion, and its mobility varies; usually, however, it is movable with respiration. The author recommends puncture with a sharp cannula of thin lumen in cases of doubt. Once the diagnosis has been made the only treatment is surgical, and the tumour may either be extirpated, a method which is beset with difficulties, or a splenectomy undertaken. Total extirpation of the spleen in such cases is usually attended with good results.

